# Atlas of Human Skeleton Hardness Obtained Using the Micro‐indentation Technique

**DOI:** 10.1111/os.12841

**Published:** 2021-05-11

**Authors:** Sheng Li, Jian‐zhao Wang, Bing Yin, Zu‐sheng Hu, Xiao‐juan Zhang, Wei Wu, Guo‐bin Liu, Ya‐ke Liu, Lei Fu, Ying‐ze Zhang

**Affiliations:** ^1^ The Third Hospital of Hebei Medical University Shijiazhuang China; ^2^ Key Biomechanics Lab of Hebei Province Shijiazhuang China

**Keywords:** Human skeleton, Implant Design, Indentation, Mechanical Properties, Microhardness

## Abstract

**Objectives:**

Measure and systematically evaluate the distribution of microhardness in the human skeleton.

**Methods:**

Three fresh corpses were obtained, aged 62 (male), 45 (female), and 58 years (male). Soft tissues were removed, and all axial and unilateral appendicular bones were freshly harvested. All three skeletons were examined by X‐ray and computed tomography (CT) to exclude skeletal pathology. Only bones from donors with no known skeletal pathology were included in the study. Axial and unilateral appendicular skeleton bones from each of the three donors were obtained, except for ear ossicles, hyoid bone, tailbone, and 14 phalanges of the foot, for which samples were difficult to obtain. Precision bone specimens with a thickness of 3 mm, which were cut with a Buehler IsoMet 11‐1280‐250 low‐speed diamond saw (Buehler, USA), were obtained from all important anatomic sites in a direction perpendicular to the mechanical axis of each bone. Micro‐indentation (the Vickers hardness test) was performed on the surface of each specimen using a microhardness tester with a diamond indenter. Hardness value (HV) was computed for each indentation. Each bone specimen was divided into several regions of interest. Indentations were carefully made and computed. Then we analyzed the data to identify hardness distribution rules at different anatomic sites.

**Results:**

In total, 5360 indentations were made in 1072 regions of interest in each donor. Hardness of the axial and appendicular bones were all inhomogeneous depending on the anatomic sites, but the distribution of microhardness followed certain rules. The mean hardness value ranged from 24.46 HV (HV = hardness value, kgf/mm^2^) for the sacrum to 53.20 HV for the shaft of the tibia. The diaphysis was harder than the metaphysis, and the proximal and distal epiphysis had lower values (8.85%– 40.39%) than the diaphysis. Among the long bone diaphyses, the tibia cortical bone (51.20 HV) was the hardest, harder than the humerus (47.25 HV), the ulna (43.26 HV), the radius (42.54 HV), and the femur (47.53 HV). However, in some anatomic sites such as the lumbar vertebra (cortical bone 32.86 HV, cancellous bone 31.25 HV), the cortical shells were sometimes not harder than the internal cancellous bones. The lumbar vertebra (32.86 HV) was harder than the cervical vertebra (28.51 HV) and the thoracic vertebra (29.01 HV).

**Conclusions:**

The distribution of microhardness in the human skeleton follows certain rules. These distribution rules could be used to predict the mechanical properties of bone and progress in this field could provide data for the basis of a new three‐dimensional printing technique, which may lead to new perspectives for custom‐made implants.

## Graphical abstract

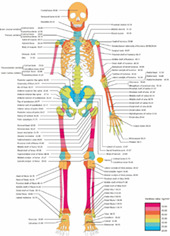

The data of bone microhardness, which represents bone material mechanical properties, is inhomogeneous among anatomical regions, but the distribution of microhardness follows certain rules. Hardness values could be used to predict the mechanical properties of bone; if the region has a higher hardness value it may provide better support and anchorage force for the physiological load or implants. Progress in this field could provide data for the basis of a new three‐dimensional printing technique and may lead to new perspectives for custom‐made implants.

## Introduction

Bone is an anisotropic and inhomogeneous composite and has an ordered structure. Systemic understanding of the distribution of the mechanical properties of all parts of the skeleton is important, because it can help us understand the mechanism of fractures or to design new types of implants with varying moduli of elasticity. The characterization of the distribution of bone mechanical properties could also serve as the theoretical basis for the design of three‐dimensional (3D) printing implants. However, holistic understanding is difficult because bone mechanical properties are co‐regulated by both the genotype and epigenetic factors^1^.

Bone has hierarchical structures with important characteristics at the millimeter scale; nevertheless, it is mechanically anisotropic and complex at small dimensions^2^. All these traits make it difficult to measure mechanical properties accurately in small anatomic sites. There are many traditional methods to evaluate the mechanical properties of bone, such as tensile, compressive, three‐ or four‐point bending, and torsion tests, the results of which are used to create stress–strain curves and to describe the properties of bones^3^. However, the specimen required for these methods must be sufficiently large, and too small a specimen results in inaccurate measurement. There are also indirect methods that can be used: for instance, ultrasonic testing, scanning acoustic microscopy, and finite element analysis. Each of these methods has advantages and disadvantages.

Several published studies have examined the mechanical behavior of human bone from different anatomic sites. Goldstein^4^ found that the mechanical properties of trabecular bone are dependent on anatomic location and function; Elise *et al*. tested the yield strain^5^ and the modulus of trabecular bone from various anatomic sites; Bayraktar *et al*.^6^ compared the elastic and yield properties of the femur; Helgason *et al*.^7^, in their respective research, analyzed the factors of mechanical properties. Unfortunately, a comprehensive evaluation of the micro‐scale level mechanical properties of bone tissue from all important anatomic sites of the human body remains unavailable.

In contrast with traditional methods, the micro‐indentation technique is well‐suited to examine local mechanical properties in inhomogeneous bone material^8^, and only a small volume of samples are required for each indentation.

Hardness is described as the resistance to plastic deformation; it is defined as the applied load divided by the residual indentation area. Bone microhardness quantified through the micro‐indentation technique could be used as an indicator of the mechanical properties of bone material. It is not a single mechanical property but integrates all constitutive behaviors of a material exhibited during deformation^9^. It is believed that bone hardness measured using the Vickers hardness test is an important methodology for the evaluation of bone mechanical properties at the level of the bone structural unit (BSU). Nanoindentation has become increasingly popular in recent years. However, in some cases, the dimensional scale of nanoindentation may be too small (at the lamellar level)^10^; in contrast to nanoindentation for assessing parameters at the lamellar level, microhardness measured by micro‐indentation appears most suited to testing mechanical properties of bone at the BSU level. Many scholars have conducted detailed work on bone microhardness^11^, ^12^. However, these studies have only focused on certain bones or anatomic sites and the results are scattered and incomparable. A comprehensive and systematic evaluation of the microscale‐level mechanical properties of bone tissue from all important anatomic sites of the human skeleton remains unavailable. The micro‐indentation technique was chosen in the present study because it is non‐destructive and allows for the repeated examination of small and awkward structures. In addition, it can provide data on the anisotropy of the regional heterogeneity and material properties near the interface^13^.

Today, 3D printing is being used to produce custom‐made implants or scaffolds to reconstruct a bone substitute; however, the 3D printing technique is generally based on the geometric and structural information derived from imaging examinations, without considering the difference in material properties. Materials applied to 3D printing implants need to be suitable for load‐bearing and the induced strain of the implants. Specially designed or custom‐made implants based on the hardness atlas may be helpful to make new medical advances. In this study, we used the micro‐indentation technique to derive the distribution of bone hardness, and by analyzing the data collected from the measurement, we aimed to investigate the rule that governs the distribution of hardness in the human skeleton.

## Methods

### Sample Preparation

Three fresh corpses (of Chinese origin, from the Hebei Province) were obtained from the Anatomy Department of the Hebei Medical University (Shijiazhuang, Hebei, China). The study was approved by the ethics committee of the Third Hospital of the Hebei Medical University and registered on the World Health Organization International Clinical Trials Registry Platform (ICTRP). The three donors' ages were 62 (male), 45 (female), and 58 years (male). All three corpses were examined by X‐ray and quantitative CT to exclude skeletal pathology. Soft tissues were removed, and all axial and unilateral appendicular bones were freshly harvested except the ear ossicles, the hyoid bone, the tailbone, and the 14 phalanges of the foot, from which obtaining samples were difficult. All the bones were stored at −20°C.

**Fig. 1:**
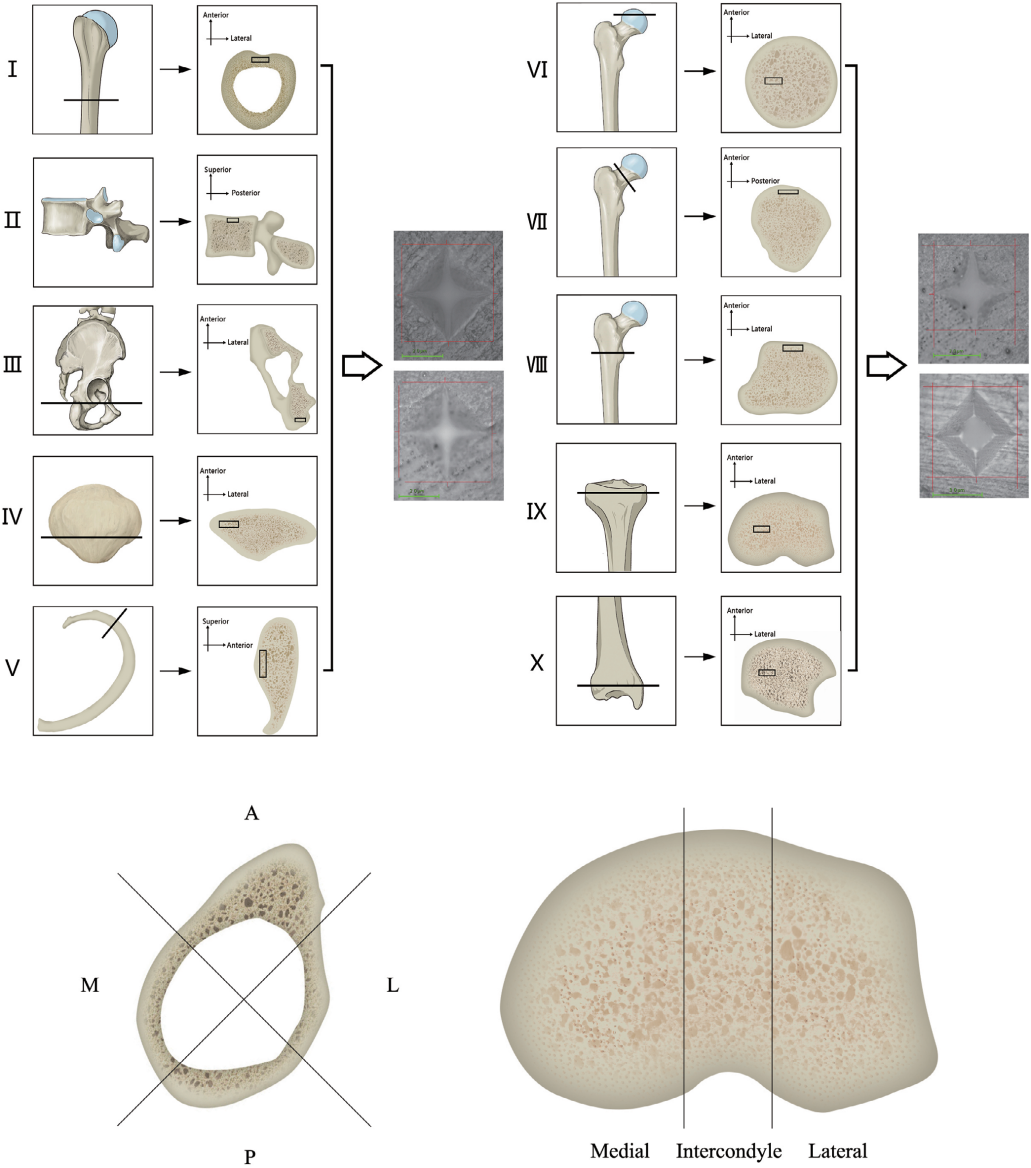
Preparation of the specimens and hardness measurements. Locations where specimens were obtained and the method of choosing regions of interest (ROI) are shown here. (I) Shaft of the humerus. (II) Spine. (III) Pelvis. (IV) Patella. (V) Rib. (VI–VIII) Femur. (IX–X) Tibia. Each specimen is 3 mm thick and ROI were chosen depending on anatomic sites. In the diaphysis of long bones, the cross‐section of the diaphysis was divided into four ROI: anterior, posterior, medial, and lateral (all of which were measured). In the tibial plateau, the cross‐section was divided into three ROI: medial, intercondylar, and lateral.

Preliminarily, each bone was sawed using a 10" band saw into several parts to facilitate further precise cutting. Precision cuts were conducted with a Buehler IsoMet 11‐1280‐250 low‐speed diamond saw (Buehler, USA). Figure 1 presents some of the locations from where samples were obtained. Bone specimens with a thickness of 3 mm were obtained from all important anatomic sites in a direction perpendicular to the mechanical axis of each bone.

After being fixed on a glass sheet with epoxy resin, the specimens were polished using sandpaper, switching progressively to finer sandpaper down to 2000 grit. A constant stream of water was used to cool the samples during all cutting and polishing operations.

### Micro‐indentation Test

Hardness is described as the resistance to penetration and plastic deformation and is defined as the applied load divided by the residual indentation area. Micro‐indentation (the Vickers hardness test) was performed using a microhardness tester (Model KB5BVZ‐Video, Germany) with a Vickers diamond indenter, and the units were measured as hardness value (HV) or kgf/mm^2^. Each bone specimen was divided into several regions of interest (ROI). A minimum of five effective indentations were performed randomly in each ROI on the surface of the sample. Figure 1 shows the method for choosing ROI in different bones. Indentations were carefully made with a distance of at least five diagonals in length from each other to avoid any deformation of neighboring indentations. The indentations for which one diagonal was 10% longer or more than the other were considered invalid and ignored^14^. The mean value of these five effective values is recorded as the hardness value of this ROI. According to the standard test method from the American Society for Testing and Materials and previous studies^15^, ^16^, the procedure was defined by a load of 50 gf, the indentation time was set to 50 s, and the dwell time was set to 12 s. The hardness value (HV) was computed for each indentation.

### Hardness Value

As the main observed indicator of this study, the Vickers hardness value is the quotient obtained by dividing the kgf load by the square mm area of indentation. Vickers hardness value is used to represent the material property and the mechanical characteristics of the bone at microstructural scale.

### Statistical Analysis

The statistical analysis aimed to determine differences among the hardness values measured at different ROI and anatomic sites. Microhardness distribution was compared across sites using *t*‐tests or one‐way analysis of variance followed by Scheffe and Tukey honestly significant difference *post hoc* tests (SPSS, Version 22; IBM SPSS, NY, USA). Normality was tested using the Shapiro–Wilk test. If data were not normally distributed or the equal variance test failed, the Mann–Whitney *U*‐test or the Kruskal–Wallis test was used. Alpha was set to *P* < 0.05 and considered statistically significant.

## Results

**Fig. 2:**
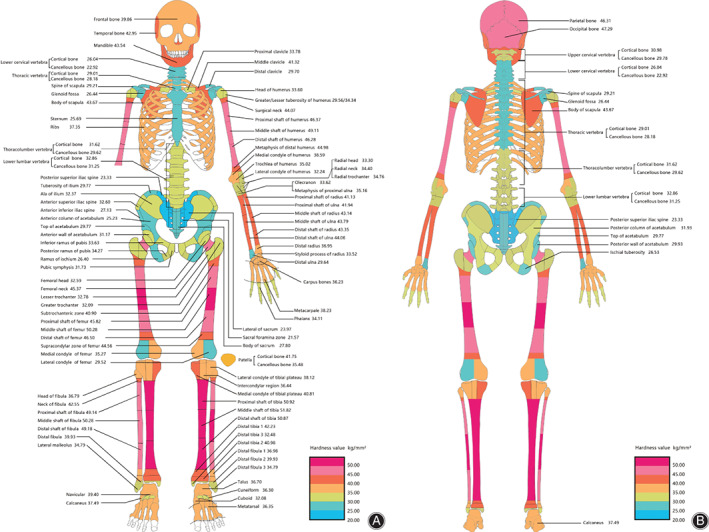
Gradient distribution of microhardness. (A) Anterior view.

In total, 5360 indentations were made in 1072 ROI in each donor. The general distribution of hardness is shown in Fig. 2. The hardness of bone was inhomogeneous among different anatomic sites across the whole skeleton. The mean hardness value ranged from 21.57 HV (HV = hardness value, kgf/mm^2^) for the sacrum to 51.82 HV for the shaft of the tibia.

In brief, the hardness of cortical bone and cancellous bone from axial and appendicular bones was inhomogeneous depending on the anatomic sites. The differences among the three donors were not statistically significant (appendicular cortical bones *P* = 0.175, axial bones *P* = 0.850, appendicular cancellous bones *P* = 0.142).

Among the axial bones, except for the skull bones where hardness values were all more than 40 HV, the ribs were the hardest, followed by the pubis, which was harder by 42.85% than the sacrum, which was the weakest bone (*P* < 0.001). In the spine, the cortical bone in the lumbar vertebrae was harder than those of the cervical vertebrae and thoracic vertebrae (*P* = 0.001 and *P* = 0.004, respectively).

### Diaphysis and Epiphysis

In appendicular long bones, hardness values were higher in the diaphysis than the metaphysis (*P* < 0.001), and the proximal and distal epiphysis had lower values (8.85%–40.39%) than the diaphysis. However, there were not always statistical differences between the proximal and distal metaphysis bones. Furthermore, the clavicle, metacarpal, and metatarsal, which had a similar structure to long bones of one shaft and two ends (*P* < 0.001), showed a similar hardness distribution pattern. Hardness values are showed in Fig. 2.

### Cortical Bones of the Long Bone Diaphysis

Among cortical bones from the long bone diaphysis, the tibia cortical bone (51.20 HV) is the hardest, and was harder than the humerus (47.25 HV; *P* = 0.002), the ulna (43.26 HV; *P* < 0.001), the radius (42.54 HV, *P* < 0.001), and the femur 47.53 HV (*P* = 0.002). The hardness of the humeral shaft was equal to that of the femoral shaft (*P* = 0.986). The difference between the tibia and fibula (49.53 HV) was not statistically significant (*P* = 0.095), and neither was the difference between the ulnar and the radius shaft (*P* = 0.329).

### Cortical and Cancellous Bones

Although the hardness value in the diaphyseal cortical bone was significantly higher than in the metaphyseal cancellous bone, there was not always a significant difference between the cortical and adjacent cancellous bone. In some anatomic sites such as the lumbar vertebrae (cortical bone 32.86 HV, cancellous bone 31.25 HV), the cortical shells were sometimes not harder than the internal cancellous bones (*P* = 0.223).

### Vertebral Bodies

In the spine, the bone in the lumbar vertebrae (32.86 HV) was harder than those of the cervical vertebrae (28.51 HV) and the thoracic vertebrae (29.01 HV; *P* < 0.001 and *P* = 0.004, respectively). Q19.

## Discussion

We examined bone microhardness in the most important anatomic sites of the human skeleton in three Chinese donors. Several interesting observations can be made with respect to the distribution of hardness. Microhardness is inhomogeneous all over the skeleton. The highest hardness value was seen in the tibial shaft and the lowest was seen in the sacrum.

### Factors of the Differences in Bone Hardness Distribution

Even within bones with the same tissue type, hardness values varied within anatomic sites. In the whole skeleton, hardness values vary among anatomic sites. These results may suggest that the distribution of hardness is regulated by some intrinsic factors and is not simply affected by tissue type. Jepsen *et al*.^17^ found that bone morphologic and tissue quality traits are co‐regulated to satisfy daily loading demands. However, bone traits are not regulated by the same set of genes, and Judex *et al*.^18^ found that the genetic control of bone morphology and tissue‐level traits are highly site specific, even within a given bone. In brief, bone hardness is regulated by genetic factors, but the regulation process is complicated and influenced by non‐genetic factors.

Recent studies have considered cortical bones and the adjacent cancellous bones as two different materials. Guo and Goldstein^19^ carefully tested Young's modulus of cancellous and cortical bones, and found that Young's moduli are quite different. Rho *et al*.^2^ considered that the remodeling rate of cancellous bone is faster than that of cortical bone, which led to the difference in mechanical properties. In our study, we found that in long bones, the mean hardness of cancellous bones is 8.85%–40.39% lower than in cortical bone on average (*P* < 0.05), and we believe that there is not only a structural difference between the two types of bone but that a difference in bone material exists as well.

### 3D Printing and Hardness Distribution

The results of our study could be applied to finite element modeling and 3D prosthesis design. In recent years, the 3D printing technique has been applied to produce custom‐made implants^20^ or scaffolds to reconstruct a bone substitute^21^; however, the 3D printing technique is generally based on the geometric and structural information derived from imaging examinations, without concern for the difference in bone material properties. Materials applied to 3D printing should not be homogeneous, and they must tolerate local stress to avoid invasive strain and the effects of stress shielding. When implants made by homogeneous materials are exposed to non‐uniform stress, microdamage induced by repetitive load accumulates in the high‐strain area, eventually leading to unexpected failure. Ideal implants should have a morphology and mechanical properties of the materials similar to natural bones at different anatomic sites to satisfy load‐bearing and stress transmission. The data collected from our study could contribute to the design of advanced or custom‐made implants, or even artificial bones.

### Limitations of the Study

The limitations of the study include the small sample size and that classification was not differentiated by age or gender.

## Conclusion

The data presented in our study suggests that bone microhardness, which represents bone material mechanical properties, is inhomogeneous among anatomical regions, but the distribution of microhardness follows certain rules. Hardness values could be used to predict the mechanical properties of bone; if the region has a higher hardness value it may provide better support and anchorage force for the physiological load or implants. Progress in this field could provide data for the basis of a new 3D printing technique and may lead to new perspectives for custom‐made implants.

## References

[1] Eckstein F , Faber S , Mühlbauer R . Functional adaptation of human joints to mechanical stimuli. Osteoarthr Cartil, 2002, 10: 44–50. 10.1053/joca.2001.0480 11795982

[2] Rho JY , Kuhn‐Spearing L , Zioupos P . Mechanical properties and the hierarchical structure of bone. Med Eng Phys, 1998, 20: 92–102. 9679227 10.1016/s1350-4533(98)00007-1

[3] Turner CH , Burr DB . Basic biomechanical measurements of bone: a tutorial. Bone, 1993, 14: 595–608. 8274302 10.1016/8756-3282(93)90081-k

[4] Goldstein SA . The mechanical properties of trabecular bone: dependence on anatomic location and function. J Biomech, 1987, 20: 1055–1061. 3323197 10.1016/0021-9290(87)90023-6

[5] Morgan EF , Keaveny TM . Dependence of yield strain of human trabecular bone on anatomic site. J Biomech, 2001, 34: 569–577. 11311697 10.1016/s0021-9290(01)00011-2

[6] Bayraktar HH , Morgan EF , Niebur GL . Comparison of the elastic and yield properties of human femoral trabecular and cortical bone tissue. J Biomech, 2004, 37: 27–35. 14672565 10.1016/s0021-9290(03)00257-4

[7] Helgason B , Perilli E , Schileo E . Mathematical relationships between bone density and mechanical properties: a literature review. Clin Biomech, 2008, 23: 135–146. 10.1016/j.clinbiomech.2007.08.024 17931759

[8] Oyen ML . Nanoindentation hardness of mineralized tissues. J Biomech, 2006, 39: 2699–2702. 16253265 10.1016/j.jbiomech.2005.09.011

[9] Hoffler CE , Moore KE , Kozloff K . Heterogeneity of bone lamellar‐level elastic moduli. Bone, 2000, 26: 603–609. 10831932 10.1016/s8756-3282(00)00268-4

[10] Zioupos P . In vivo fatigue microcracks in human bone: material properties of the surrounding bone matrix. Eur J Morphol, 2005, 42: 31–42. 16123022 10.1080/09243860500095463

[11] Weaver JK . The microscopic hardness of bone. J Bone Joint Surg Am, 1966, 48: 273–288. 5932913

[12] Öhman C , Zwierzak I , Baleani M . Human bone hardness seems to depend on tissue type but not on anatomical site in the long bones of an old subject. Proc Inst Mech Eng Pt H J Eng Med, 2013, 227: 200–206. 10.1177/0954411912459424 23513991

[13] Zioupos P , Currey JD , Casinos A . Exploring the effects of hypermineralisation in bone tissue by using an extreme biological example. Connect Tissue Res, 2000, 41: 229–248. 11264871 10.3109/03008200009005292

[14] Ziv V , Wagner HD , Weiner S . Microstructure‐microhardness relations in parallel‐fibered and lamellar bone. Bone, 1996, 18: 417–428. 8739899 10.1016/8756-3282(96)00049-x

[15] ASTM Standard . Standard Test Method for Microindentation Hardness of Materials. ASTM International, 2017, E384, 1–40.

[16] Dall'Ara E , Schmidt R , Zysset P . Microindentation can discriminate between damaged and intact human bone tissue. Bone, 2012, 50: 925–929. 22270054 10.1016/j.bone.2012.01.002

[17] Wallace IJ , Tommasini SM , Judex S . Genetic variations and physical activity as determinants of limb bone morphology: an experimental approach using a mouse model. Am J Phys Anthropol, 2012, 148: 24–35. 22331623 10.1002/ajpa.22028

[18] Judex S , Garman R , Squire M . Genetically Based Influences on the Site‐Specific Regulation of Trabecular and Cortical Bone Morphology. J Bone Miner Res, 2004, 19: 600–606. 15005847 10.1359/JBMR.040101

[19] Guo XE , Goldstein SA . Vertebral trabecular bone microscopic tissue elastic modulus and hardness do not change in ovariectomized rats. J Orthop Res, 2000, 18: 333–336. 10815837 10.1002/jor.1100180224

[20] Bergmann C , Lindner M , Zhang W . 3D printing of bone substitute implants using calcium phosphate and bioactive glasses. J Eur Ceram Soc, 2010, 30: 2563–2567.

[21] Bose S , Vahabzadeh S , Bandyopadhyay A . Bone tissue engineering using 3D printing. Mater Today, 2013, 16: 496–504.

